# African Immigrant Mothers’ Views of Perinatal Mental Health and Acceptability of Perinatal Mental Health Screening: Quantitative Cross-sectional Survey Study

**DOI:** 10.2196/40008

**Published:** 2023-01-27

**Authors:** Chinenye Nmanma Nwoke, Oluwagbohunmi A Awosoga, Sheila McDonald, Glenda T Bonifacio, Brenda M Y Leung

**Affiliations:** 1 Faculty of Health Sciences University of Lethbridge Lethbridge, AB Canada; 2 Department of Pediatrics and Community Health Sciences Faculty of Medicine University of Calgary Calgary, AB Canada; 3 Department of Women and Gender Studies University of Lethbridge Lethbridge, AB Canada

**Keywords:** African women, perinatal mental health, screening, anxiety, mental health literacy, pregnancy, postpartum, depression, acceptability, knowledge

## Abstract

**Background:**

Mental health disorders are the most common perinatal conditions. They affect mothers, babies, partners, and support networks. However, <15% of pregnant and postpartum women seek timely help for their mental health care. Low perinatal mental health knowledge and universal screening unacceptability are cited as important deterrents to obtaining timely mental health care.

**Objective:**

The purpose of this quantitative cross-sectional study was 2-fold: (1) to determine African immigrant mothers’ views of perinatal mental health and to identify predictors of those views and (2) to identify African immigrant mothers’ views regarding perinatal mental health screening and to determine factors associated with those views.

**Methods:**

A cross-sectional survey was conducted using a convenience sample of African immigrant women from the province of Alberta, Canada. Respondents were eligible to participate if they were aged ≥18 years, had a live birth, and the infant was aged ≤2 years. Questions were drawn from the Edinburgh Postnatal Depression Scale, the Generalized Anxiety Disorder-7 scale, and additional questions were developed using the Alberta Maternal Mental Health 2012 survey as a guide and tested to reflect the immigrant context. Descriptive and multivariable regression analyses were conducted.

**Results:**

Among the 120 respondents, 46.5% (53/114) were aged 31-35 years, 76.1% (89/117) were employed or on maternity leave, 92.5% (111/120) were married, and 55.6% (65/117) had younger infants aged 0 to 12 months. Significantly more respondents had higher levels of knowledge of postnatal (109/115, 94.8%) than prenatal (57/110, 51.2%) mental health (*P*<.001). Only 25.4% (28/110) of the respondents accurately identified that prenatal anxiety or depression could negatively impact child development. Personal knowledge of postpartum anxiety and depression was a significant predictor of prenatal and postnatal mental health knowledge. Most respondents strongly agreed or agreed that all women should be screened in the prenatal (82/109, 75.2%) and postnatal (91/110, 82.7%) periods. Respondents reported that their partner would be their first choice when seeking help and support. The acceptability of postnatal screening was a significant predictor of prenatal mental health knowledge (*P*<.001), whereas the acceptability of prenatal screening was a significant predictor of postnatal mental health knowledge (*P*=.03). Prenatal mental health knowledge was a significant predictor of both prenatal (*P*<.001) and postnatal (*P*=.001) screening acceptability.

**Conclusions:**

Although African mothers’ knowledge of postnatal mental health is high, their prenatal mental health knowledge and its influence on child development are limited. Perinatal mental health interventions for African immigrant mothers in Alberta should target these knowledge gaps. The high acceptability of universal perinatal mental health screening among African mothers provides a promising strategy for perinatal mental health literacy initiatives to achieve optimal perinatal mental health.

## Introduction

### Background

Depression and anxiety are the most common mental health conditions experienced by pregnant and postpartum women, with a prevalence ranging from 8% to 25% [1-3]. For immigrants, their postpartum maternal mental health appears to be particularly poor; specifically, the occurrence and associated risks of depression and anxiety appear to be twice as high in recent immigrant women when compared with their Canadian counterparts [4]. Maternal depression and anxiety in the period after childbirth have been shown to have negative consequences [4,5]. For immigrants, these consequences could be much greater because of other vulnerabilities associated with the stressful life event of migration to a new country [5].

A review study showed that maternal depression has the potential to affect not only mothers but also their partners, babies, and support networks (ie, other family members) [6]. For mothers, the potential consequences of depression could include breastfeeding for shorter periods and the use of alcohol, cigarettes, or other substances that are harmful to the baby [7,8]. Depressed women are also at an increased risk of future episodes of more severe mental health disorders, including suicide, outside the postpartum period, especially in untreated instances [9,10]. Moreover, these women may have negative views of motherhood and of themselves as mothers, see their baby’s behavior as *difficult*, and may not recognize their baby’s cues to respond appropriately [11,12]. For babies, the potential consequences of maternal depression and anxiety include behavioral disturbances; for example, babies are prone to cry often, louder, and in bouts of time and spend less time in the “quiet and alert” state when they learn the most about their environment [13-15]. These babies may have developmental delays, as they may walk and talk later than others [13]. Finally, for partners, the potential consequences of maternal depression and anxiety may include relationship disruption (ie, increased risk of separation or divorce) and sympathetic depression (the partners themselves also experience depression), resulting in the need to treat the partners [16,17]. Surprisingly, other studies have shown that partners experiencing depression in the postpartum period experience many of the same negative impacts on their relationships and their babies, similar to that seen in the instances of depression and anxiety in mothers [6,18]. Nonetheless, in the case of immigrants, these aforementioned effects on mothers, babies, and partners have the potential to worsen further [6].

Moreover, 48% of mothers with anxiety and 70% with depression will continue to experience symptoms after delivery [19-21] and throughout their children’s early years of life [22,23] if they do not receive adequate treatment. Despite this, only a small percentage of pregnant and postpartum women actively seek help or treatment options, and <15% access mental health services [24,25]. A Canadian study found that immigrants from a visible minority population (ie, racialized immigrants) were less likely to consult with mental health professionals; the stressors of migration and resettlement can have profound mental health implications, including mental health professional consultations [26]. Although system-level barriers (ie, long wait times, language barriers, lack of culturally safe care, the proximity of mental health services and difficulties with transportation, and challenges in finding timely childcare) limit access to mental health services, personal barriers (ie, views of mental health and its treatment, help-seeking preferences) are also cited as significant barriers to mental health care [25,26] and impact help-seeking outcomes and adherence to treatment [27,28].

Studies have shown that most Africans tend to cope with depression and anxiety by using informal resources such as the church and their family [5,29]. However, there is a lack of literature on whether these informal coping mechanisms used by Africans are effective in decreasing the burden or associated risk factors of maternal depression and anxiety. A review of other literature highlighted the fact that most African women, when compared with White women, held stronger beliefs that “problems” should not be discussed outside the family, including with neighbors, friends, and health professionals [29-32]. These “problems” could sometimes include maternal mental illnesses, such as depression and anxiety. African immigrants were more likely to seek help from ministers in a church setting; if a minister was contacted first, the likelihood of seeking help from other sources (eg, health professionals) could decrease [4,29].

Seeking help from a church minister or family member is suitable for personal family issues and in some cases health concerns as well [29]. However, for maternal depression and anxiety, it could become unfitting in instances when adequate or appropriate referrals and support are not provided in a timely manner [33]. Not seeking the right help at the right time could possibly move the individual further down the continuum of mood and anxiety disorders, worsening the condition to a more advanced stage of illness, which is the stage when most immigrants present themselves to health professionals or the health care system in Canada [4]. Maintaining the stereotypical image of the self-reliant and strong African woman also often hinders seeking appropriate help for depression and anxiety [4,29]. There is a need for more research into how views on perinatal mental health and universal screening acceptability impact maternal mental health among African mothers in Alberta.

Having a better understanding of African mothers’ views on depression and anxiety in the perinatal period and views on the acceptability of screening provides a valuable basis for understanding the mental health decision-making and health care use patterns of African immigrant mothers in the perinatal period. In addition, few studies have explored the public’s views on perinatal mental health [1,23,34], and none have done so among African immigrant mothers in Canada.

### Objective

The purpose of this study was to examine how African immigrant mothers understood the topics related to the knowledge of prenatal and postnatal mental health and their influence on maternal and child outcomes, views on screening acceptability, and to determine factors associated with perinatal mental health knowledge and screening acceptability.

## Methods

### Participants

This cross-sectional study was conducted from January 2020 to December 2020 in Alberta, Canada. Detailed recruitment strategies for this study are given elsewhere [35]. We recruited 120 African immigrant mothers aged ≥18 years, with infants aged ≤2 years. Participants were identified and recruited in partnership with community organizations, immigrant-serving organizations, religious leaders, and snowballing recruitment strategy. The inclusion criteria were as follows: the participant must (1) be aged ≥18 years; (2) be a resident of Alberta; (3) have had a live birth, with the infant aged ≤2 years; and (4) self-identify as African. Respondents who answered ‘yes’ to all 4 questions were deemed eligible to participate in the study. Following this, the participants were able to access the 63-item study questionnaire on the web through the Qualtrics platform (version 2020; Qualtrics) or request for a paper questionnaire. Questions were drawn from the Edinburgh Postnatal Depression Scale and the Generalized Anxiety Disorder-7 scale; additional questions were developed using the Alberta Maternal Mental Health 2012 survey as a guide and tested to reflect the immigrant context. Participants selected responses to items related to perinatal mental health on a 5-point Likert scale ranging from strongly disagree to strongly agree. All the questions were pilot tested, and informed consent was obtained before questionnaire administration (ie, data collection).

### Survey Administration

The study questionnaire was programmed using the Qualtrics survey platform. Before data collection, informed consent was obtained from the prospective study participants through the Qualtrics survey platform. All the participants were advised that they had the right to withdraw from the study at any time without any consequences; this was explicitly noted in the letter of consent. Participants who met the study inclusion criteria and provided consent were automatically able to access the study questionnaire via a link to Qualtrics. The prelude to the questions included the study rationale in layman’s terms, description of the survey structure and indicators of survey progress (using static statements), anticipated time commitment, and the researcher’s contact information. The survey was made accessible to eligible participants until the end of the study recruitment period. The Qualtrics platform allows partially completed survey data to be saved; thus, participants were able to save and continue the survey if any interruptions arose.

The option of having a paper-based version of the study questionnaire mailed to the participants’ residence was offered to respondents with no access to the internet. These packages included the recruitment memo outlining the purpose of the study, a paper-based version of the study consent form, a paper-based version of the study questionnaire, and a list of resources and support services from Alberta Health Services available to mothers in Alberta. Participants were provided with a postage-paid return envelope and were asked to return the questionnaire package at their earliest convenience. If the questionnaire package was not returned after 3 weeks, reminders were sent via the respective method that the respondent had expressed interest.

By using the primary method of survey administration for this study (ie, on the web via Qualtrics) in addition to providing alternative administration options at community and cultural events and having a paper-based version mailed out, the study was able to reach a suitable sample size of 120 African immigrant mothers.

### Pretesting

The questionnaire was pretested with 5 respondents who were not part of the total sample size to ascertain its content and face validity before administering it to the target population. An important goal of the pilot was to assess response latency, that is, the amount of time it took to complete individual sections and the full study questionnaire, response wording, logical flow, and the overall impression of the survey. Minor issues were identified and resolved based on their comments and feedback. Overall, 2 (40%) of the 5 respondents had difficulty in understanding a few terms (eg, the definition of an immigrant). To rectify this issue, detailed definitions were provided for all terminologies used in the final study questionnaire. Nonetheless, pilot testing revealed that the remaining questions on the survey were suitable for the study population (African immigrant mothers). During the pilot testing, respondents took on average 10 to 15 minutes to complete the questionnaire, and thus the response latency time was deemed appropriate.

### Outcomes

The main outcomes were (1) knowledge of prenatal and postnatal mental health and its adverse consequences and (2) African mothers’ views related to universal prenatal and postnatal mental health screening. Prenatal and postnatal mental health were defined as anxiety or depression occurring in the prenatal and postnatal periods, respectively.

For each outcome of knowledge, the dichotomized definition of “high” and “low” knowledge was based on the level of agreement to statements. A high level of prenatal mental health knowledge was defined by a response of strongly or somewhat agree to at least one of the 2 following questions: “Women who have had anxiety or depression in the past (before they became pregnant) are more likely to experience anxiety or depression when they are pregnant;” and/or “Women who have anxiety or depression during pregnancy are more likely to experience postpartum depression,” while those with low prenatal knowledge answered strongly or somewhat disagree to either or both questions. High postnatal knowledge was defined as a response of strongly or somewhat agree to at least one of 3 following questions: “Women who have postpartum depression find it more difficult to respond to their baby’s cues;” “Women who have postpartum depression find it more difficult to respond to the needs of their partner and other children;” and “Partners of women who have postpartum depression are also at risk for depression;” whereas those with low postnatal knowledge responded strongly disagree or somewhat disagree to one or more of these questions. Views of prenatal screening were considered acceptable if participants responded to the item “All women should be screened for depression and anxiety in pregnancy” with a response of strongly agree or agree (vs strongly disagree, disagree, or neither agree nor disagree). A similar item related to postnatal screening assessed its acceptability.

### Data Analysis

Descriptive data consisted of frequencies, percentages, and chi-square or Fisher exact test to determine differences between categorical variables. Cramer V (an adjusted version of the chi-square test) was used to determine the association between 2 categorical variables. The strength of the association was interpreted through effect size. Unadjusted odds ratios and 95% CIs were calculated for each independent variable and the outcome variables (pre- and postnatal mental health knowledge; acceptability of pre- and postnatal mental health screening). Variables associated with the outcomes at a level of *P*<.20 in the unadjusted analyses were considered for inclusion in the multivariable models. Correlations among independent variables that met the criteria for model entry were generated to assess multicollinearity (*r*>0.4). Four multivariable logistic regression models were constructed with all the variables entered simultaneously to identify predictive variables significantly associated with the study outcomes, which were presented as adjusted odds ratios and 95% CIs. Statistical significance was set at *P*<.05. Model robustness was assessed by entering the excluded variables back into the final models and computing the Akaike information criterion to determine the best-fit model. All analyses were performed using SAS Enterprise (version 7.1; SAS Institute Inc).

### Ethics Approval

The University of Lethbridge Human Participant Research Committee approved this study on December 11, 2019 (protocol number 2019-116). Participants were provided with a study information sheet and were asked to sign a consent form before accessing the study questionnaire.

## Results

### The Sample

The age of the respondents ranged from 24 to 40 (mean 31.7, SD 3.2) years, and 46.5% (53/114) of respondents were aged between 31 and 35 years. Of the proportion of African immigrant women with infants aged ≤2 years in Alberta, majority had younger infants aged 0-12 months (65/117, 55.6%), had a total household income of ≥CAD $60,000 (US $45,000; 65/116, 56%), were employed or on maternity leave (89/117, 76.1%), rented their home (78/118, 66.1%), were married (111/120, 92.5%), and had an advanced degree (masters or professional degree; 58.5%). Of note, most (69/118, 58.5%) African immigrant women in this study were highly educated with advanced degrees; however, approximately one-quarter of the respondents were unemployed (28/117, 23.9%; Table 1).

Approximately half (57/110, 51.8%) of the respondents had high knowledge levels regarding prenatal mental health, and >94% (109/115) had high levels of postnatal mental health knowledge (Table 2). The acceptability of prenatal mental health screening was high among 75.2% (82/109) of the respondents, whereas the acceptability of postnatal mental health screening was high among 82.7% (91/110) of the respondents (Table 2). Most respondents noted that if depressed during pregnancy or after having a baby, their first choice of help would be their partner (45/108, 41.7%), health care professional (36/108, 33.3%), friend or relative (25/108, 23.1%), and no one (2/108, 1.9%; Table 2).

**Table 1:** Description of study respondents (N=120).

Variable			Participants, n^a^ (%)
**Maternal age (years)**			
	18-30	47 (41.2)	
	31-35	53 (46.5)	
	≥36	14 (12.3)	
**Age of the most recent infant (months)**			
	0-12	65 (55.6)	
	<12	52 (44.4)	
**Religion**			
	Christian	109 (93.2)	
	Islam	8 (6.8)	
**Total household income (CAD $)**			
	≤59,999 (US $44,999) or less	51 (44)	
	≥60,000 (US $45,000) or more	65 (56)	
**Employment status**			
	Employed	51 (43.6)	
	Maternity leave	38 (32.5)	
	Unemployed	28 (23.9)	
**Home**			
	Own	40 (33.9)	
	Rent	78 (66.1)	
**Maternal education status**			
	Certificate or diploma or less	7 (5.9)	
	Bachelor’s degree	42 (35.6)	
	Advanced degree (>bachelor’s)	69 (58.5)	
**Marital status**			
	Married or common law	111 (92.5)	
	Single or separated or divorced	9 (7.5)	

^a^Not all variables total to N=120 owing to missing data.

**Table 2:** Summary of participant responses to perinatal mental health items (N=120).

Variable		Participants, n^a^ (%)
**Personal experience with someone who experienced postpartum anxiety or depression (n=103)**		
	Yes	53 (51.5)
	No	50 (48.5)
**Partners of women who have postpartum depression are also at risk for depression** **(n=115)**		
	Disagree	13 (11.3)
	Neither agree nor disagree	21 (18.3)
	Agree	81 (70.4)
**Women who have had anxiety or depression in the past (before they became pregnant) are more likely to experience anxiety or depression when they are pregnant** **(n=110)**		
	Strongly to somewhat disagree	30 (27.3)
	Neither agree nor disagree	23 (20.9)
	Strongly to somewhat agree	57 (51.8)
**Women who have postpartum depression find it more difficult to respond to their baby’s cues** **(** **n=110)**		
	Strongly to somewhat disagree	20 (18.2)
	Neither agree nor disagree	10 (9.1)
	Strongly to somewhat agree	80 (72.7)
**Women who have postpartum depression find it more difficult to respond to the needs of their partner and other children** **(n=110)**		
	Strongly to somewhat disagree	20 (18.2)
	Neither agree nor disagree	7 (6.4)
	Strongly to somewhat agree	83 (75.4)
**Children whose mothers were depressed or anxious during pregnancy were more likely to experience slower development** **(n=110)**		
	Strongly to somewhat disagree	31 (28.2)
	Neither agree nor disagree	51 (46.4)
	Strongly to somewhat agree	28 (25.4)
**All women should be checked for depression and anxiety during pregnancy** **(n=109)**		
	Strongly to somewhat disagree	17 (15.6)
	Neither agree nor disagree	10 (9.2)
	Strongly to somewhat agree	82 (75.2)
**All women should be checked for depression and anxiety after the baby is born** **(n=110)**		
	Strongly to somewhat disagree	15 (13.6)
	Neither agree nor disagree	4 (3.6)
	Strongly to somewhat agree	91 (82.7)
**Knowledge of prenatal mental health** **(n=110)**		
	High	57 (51.8)
	Low	53 (48.2)
**Knowledge of postnatal mental health** **(n=115)**		
	High	109 (94.8)
	Low	6 (5.2)
**Acceptability of prenatal mental health screening** **(n=109)**		
	High	82 (75.2)
	Low	27 (24.8)
**Acceptability of postnatal mental health screening** **(n=110)**		
	High	91 (82.7)
	Low	19 (17.3)
**Help-seeking behaviors** **(n=108)**		
	Partner	45 (41.7)
	Health professional	36 (33.3)
	Friend or relative	25 (23.1)
	No one	2 (1.9)

^a^Not all variables total to N=120 owing to missing data.

### Factors Associated With Prenatal and Postnatal Mental Health Knowledge

Overall, 51.8% (57/110) of all respondents strongly agreed or somewhat agreed that women with a history of anxiety or depression were more likely to experience anxiety or depression in pregnancy, whereas 27.3% (30/110) strongly disagreed or somewhat disagreed, and 20.9% (23/110) neither agreed nor disagreed (Table 2). In the final multivariable model, participants with a bachelor’s degree or less than a bachelor’s degree and who had low acceptability of postnatal screening were less likely to have high levels of knowledge of prenatal mental health (Table 3). Individuals on maternity leave were more likely to be knowledgeable about prenatal mental health than unemployed respondents (Table 3).

Regarding postnatal mental health knowledge, 72.7% (80/110) of all respondents strongly agreed or somewhat agreed that women with depression found it difficult to respond to their baby’s cues; 75.4% (83/110) strongly agreed or somewhat agreed that women with depression found it more difficult to respond to the needs of their partner or other children; and 70.4% (81/115) strongly agreed or somewhat agreed that partners of women with depression or anxiety were more likely to experience depression (Table 2). In the final multivariable model, participants who had low acceptability of prenatal screening were less likely to have high levels of postnatal mental health knowledge (Table 4). Overall, more African immigrant women had a higher knowledge of postnatal mental health (109/115, 94.8%) than that of prenatal mental health (57/110, 51.8%; *P*<.001; Table 2).

**Table 3:** Unadjusted odds ratios (UOR) and adjusted odds ratios (AOR) of factors associated with knowledge of prenatal mental health among African immigrant women in Alberta, Canada (N=120)^a^.

Variables		High level of prenatal mental health knowledge				
		Participants, n (%)	UOR (95% CI)	*P* value	AOR (95% CI)	*P* value
**Maternal age (years)**						
	≤30	41 (38.7)	0.89 (0.41-1.90)	.75	N/A^b^	N/A
	≥31	65 (61.3)	Reference	Reference	N/A	N/A
**Age of the most recent infant (months)**						
	0-12	60 (55.6)	1.73^c^ (0.80-3.73)	.16	N/A	N/A
	>12	48 (44.4)	Reference	Reference	N/A	N/A
**Religion**						
	Christian	99 (92.5)	0.42 (0.01-2.24)	.31	N/A	N/A
	Islam	8 (7.5)	Reference	Reference	N/A	N/A
**Total household income (CAD $)**						
	≥60,000 (US $45,000)	61 (57)	0.85 (0.40-1.81)	.67	N/A	N/A
	≤59,999 (US $44,999)	46 (43)	Reference	Reference	N/A	N/A
**Employment status**						
	Employed	46 (42.2)	0.88^c^ (0.34-2.30)	.09	0.68 (0.23-1.99)	.20
	Maternity leave	36 (33)	3.25^c^ (1.13-9.31)	.004	3.98^d^ (1.14-13.92)	.004
	Unemployed	27 (24.8)	Reference	Reference	Reference	Reference
**Home**						
	Rent	74 (68.5)	0.99 (0.44-2.23)	.46	N/A	N/A
	Own	34 (31.5)	Reference	Reference	N/A	N/A
**Maternal education**						
	Bachelor’s degree or less	46 (41.8)	0.48^c^ (0.22-1.04)	.06	0.36^d^ (0.14-0.92)	.03
	Advanced degree	64 (58.2)	Reference	Reference	Reference	Reference
**Marital status**						
	Married or common law	96 (91.4)	2.30 (0.54-9.70)	.26	N/A	N/A
	Not married	9 (8.6)	Reference	Reference	N/A	N/A
**Prenatal screening**						
	Not acceptable	27 (24.8)	0.10^c^ (0.03-0.32)	<.001	N/A	N/A
	Acceptable	82 (75.2)	Reference	Reference	N/A	N/A
**Postnatal screening**						
	Not acceptable	19 (17.3)	0.04^c^ (0.004-0.27)	.001	0.03^d^ (0.003-0.23)	.001
	Acceptable	91 (82.7)	Reference	Reference	Reference	Reference

^a^Some variables do not total to N=120 owing to missing responses.

^b^N/A: not applicable.

^c^Met criteria for inclusion in multivariable models (*P*<.20).

^d^Variables in the final multivariable models are adjusted for all other variables in the model. Final multivariable models present only those variables significant at *P*<.05.

**Table 4:** Unadjusted odds ratios (UOR) and adjusted odds ratios (AOR) of factors associated with knowledge of postnatal mental health among African immigrant women in Alberta, Canada (N=120)^a^.

Variables			High level of postnatal mental health knowledge								
			Participants, n (%)		UOR (95% CI)		*P* value		AOR (95% CI)		*P* value
**Maternal age (years)**											
	≤30	45 (40.5)		1.52 (0.23-8.63)		.64		N/A^b^		N/A	
	≥31	66 (59.5)		Reference		Reference		N/A		N/A	
**Age of the most recent infant** **(months)**											
	0-12	63 (55.8)		2.65 (0.47-15.11)		.27		N/A		N/A	
	>12	50 (44.3)		Reference		Reference		N/A		N/A	
**Religion**											
	Christian	104 (92.9)		—^c^		—		N/A		N/A	
	Islam	8 (7.1)		Reference		Reference		N/A		N/A	
**Total household income (CAD $)**											
	≥60,000 (US $45,000)	62 (55.4)		0.23^d^ (0.03-2.06)		.19		N/A		N/A	
	≤59,999 (US $44,999)	50 (44.6)		Reference		Reference		N/A		N/A	
**Employment status**											
	Employed	50 (43.9)		2.88 (0.45-8.38)		.31		N/A		N/A	
	Maternity leave	36 (31.6)		4.2 (0.41-7.65)		.27		N/A		N/A	
	Unemployed	28 (24.6)		Reference		Reference		N/A		N/A	
**Home**											
	Rent	77 (68.1)		2.24 (0.43-11.70)		.34		N/A		N/A	
	Own	36 (31.9)		Reference		Reference		N/A		N/A	
**Maternal education**											
	Bachelor’s degree or less	47 (40.9)		0.33 (0.06-1.86)		.70		N/A		N/A	
	Advanced degree	68 (59.1)		Reference		Reference		N/A		N/A	
**Marital status**											
	Married or common law	101 (91.8)		3.03 (0.30-30.44)		.35		N/A		N/A	
	Not married	9 (8.2)		Reference		Reference		N/A		N/A	
**Prenatal screening**											
	Not acceptable	27 (24.8)		0.30^d^ (0.06-1.61)		.10		0.09^e^ (0.01-0.77)		.03	
	Acceptable	82 (75.2)		Reference		Reference		Reference		Reference	
**Postnatal screening**											
	Not acceptable	19 (17.3)		0.18^d^ (0.03-0.98)		.02		N/A		N/A	
	Acceptable	91 (82.7)		Reference		Reference		N/A		N/A	

^a^Some variables do not total to N=120 owing to missing responses.

^b^N/A: not applicable.

^c^Zero odds in denominator, hence odds ratios are not computed.

^d^Met criteria for inclusion in multivariable models (*P*<.20).

^e^Variables in the final multivariable models are adjusted for all other variables in the model. Final multivariable models present only those variables significant at *P*<.05.

### Factors Associated With Acceptability of Prenatal and Postnatal Mental Health Screening

Approximately two-thirds (82/109, 75.2%) of all respondents somewhat agreed or strongly agreed that all women should be checked for depression and anxiety during pregnancy, with a minimal proportion (10/109, 9.2%) indicating that they neither agreed nor disagreed, and over 15% (17/109, 15.6%) indicating that they strongly disagreed or somewhat disagreed (Table 2). Similarly, most (91/110, 82.7%), respondents somewhat agreed or strongly agreed that all women should be checked for depression and anxiety after the baby is born, with a minimal proportion (4/110, 3.6%) indicating that they neither agreed nor disagreed, and over 10% (15/110, 13.6%) indicating that they strongly disagreed or somewhat disagreed (Table 2). In the final multivariable model, participants with low prenatal mental health knowledge were less likely to strongly agree or agree that all women should be screened for mental health conditions during the prenatal period (Table 5). In addition, older participants (≥31 years) and participants with low prenatal mental health knowledge were less likely to strongly agree or agree that all women should be screened for mental health conditions during the postnatal period (Table 6).

Overall, more African immigrant women had a higher acceptability of postnatal mental health screening (91/110, 82.7%) than prenatal mental health screening (82/109, 75.2%; *P*<.001). The correlation coefficient between the acceptability of prenatal and postnatal mental health screenings was high (*r=*0.745).

**Table 5:** Unadjusted odds ratios (UOR) and adjusted odds ratios (AOR) of factors associated with acceptability of prenatal mental health screening among African immigrant women in Alberta, Canada (N=120)^a^.

Variables			Acceptability of prenatal screening								
			Participants, n (%)		UOR (95% CI)		*P* value		AOR (95% CI)		*P* value
**Maternal age** **(years)**											
	≤30	41 (39.1)		1.03 (0.42-2.57)		.39		N/A^b^		N/A	
	≥31	64 (60.9)		Reference		Reference		N/A		N/A	
**Age of the most recent infant (months)**											
	0-12	59 (55.1)		1.80 (0.73-4.44)		.26		N/A		N/A	
	>12	48 (44.9)		Reference		Reference		N/A		N/A	
**Religion**											
	Christian	98 (92.5)		0.44 (0.05-3.76)		.63		N/A		N/A	
	Islam	8 (7.5)		Reference		Reference		N/A		N/A	
**Total household income (CAD $)**											
	≥60,000 (US $45,000)	60 (56.6)		1.03 (0.42-2.55)		.45		N/A		N/A	
	≤59,999 (US $44,999)	46 (43.4)		Reference		Reference		N/A		N/A	
**Employment status**											
	Employed	46 (42.6)		1.07 (0.38-3.04)		.48		N/A		N/A	
	Maternity leave	35 (32.4)		2.04 (0.61-6.80)		.21		N/A		N/A	
	Unemployed	27 (25)		Reference		Reference		N/A		N/A	
**Home**											
	Rent	73 (68.2)		2.02 (0.80-5.08)		.69		N/A		N/A	
	Own	34 (31.8)		Reference		Reference		N/A		N/A	
**Maternal education**											
	Bachelor’s degree or less	46 (42.2)		0.73 (0.30-1.74)		.47		N/A		N/A	
	Advanced degree	63 (57.8)		Reference		Reference		N/A		N/A	
**Marital status**											
	Married or common law	95 (91.4)		1.58 (0.37-6.82)		.54		N/A		N/A	
	Not married	9 (8.6)		Reference		Reference		N/A		N/A	
**Prenatal knowledge**											
	Low	53 (48.6)		0.10^c^ (0.03-0.32)		<.001		0.12^d^ (0.04-0.37)		<.001	
	High	56 (51.4)		Reference		Reference		Reference		Reference	
**Postnatal knowledge**											
	Low	6 (5.5)		0.30^c^ (0.06-1.61)		.10		N/A		N/A	
	High	103 (94.5)		Reference		Reference		N/A		N/A	

^a^Some variables do not total *to N=120owing* to missing responses.

^b^N/A: not applicable.

^c^Meets criteria for inclusion in multivariable models (*P*<.20).

^d^Variables in the multivariable model are adjusted for all other variables in the model. Multivariable models present only those variables significant at *P*<.05*.*

**Table 6:** Unadjusted odds ratios (UOR) and adjusted odds ratios (AOR) of factors associated with acceptability of postnatal mental health screening among African immigrant women in Alberta, Canada (N=120)^a^.

Variables		Acceptability of postnatal screening				
		Participants, n (%)	UOR (95% CI)	*P* value	AOR (95% CI)	*P* value
**Maternal age (years)**						
	≤30	41 (38.7)	2.94^b^ (0.91-9.55)	.07	3.94^c^ (1.10-14.08)	.03
	≥31	65 (61.3)	Reference	Reference	Reference	Reference
**Age of the most recent infant (months)**						
	0-12	60 (55.6)	1.13 (0.40-3.20)	.62	N/A^d^	N/A
	>12	48 (44.4)	Reference	Reference	N/A	N/A
**Religion**						
	Christian	99 (92.5)	—^e^	—	N/A	N/A
	Islam	8 (7.5)	Reference	Reference	N/A	N/A
**Total household income (CAD $)**						
	≥60,000 (US $45,000)	61 (57)	0.91 (0.32-2.62)	.99	N/A	N/A
	≤59,999 (US $44,999)	46 (43)	1.00	Reference	N/A	N/A
**Employment status**						
	Employed	46 (42.2)	1.95 (0.60-6.34)	.62	N/A	N/A
	Maternity leave	36 (33)	2.17 (0.61-7.79)	.46	N/A	N/A
	Unemployed	27 (24.8)	Reference	Reference	N/A	N/A
**Home**						
	Rent	74 (68.5)	2.22 (0.77-6.39)	.21	N/A	N/A
	Own	34 (31.5)	Reference	Reference	N/A	N/A
**Maternal education**						
	Bachelor’s degree or less	46 (41.8)	0.76 (0.28-2.05)	.59	N/A	N/A
	Advanced degree	64 (58.2)	Reference	Reference	N/A	N/A
**Marital status**						
	Married or common law	96 (91.4)	1.41 (0.27-7.39)	.68	N/A	N/A
	Not married	9 (8.6)	Reference	Reference	N/A	N/A
**Prenatal knowledge**						
	Low	53 (48.2)	0.04^b^ (0.01-0.27)	.001	0.03^c^ (0.004-0.24)	.001
	High	57 (51.8)	Reference	Reference	Reference	Reference
**Postnatal knowledge**						
	Low	6 (5.5)	0.18^b^ (0.03-0.98)	.12	N/A	N/A
	High	104 (94.5)	Reference	Reference	N/A	N/A

^a^Some variables do not total to N=120 owing to missing responses.

^b^Meets criteria for inclusion in multivariable models (*P*<.20).

^c^Variables in the multivariable model are adjusted for all other variables in the model. Multivariable models present only those variables significant at *P*<.05.

^d^N/A: not applicable.

^e^Zero odds in denominator, hence, odds ratios are not computed.

## Discussion

### Principal Findings

Overall, this study found that respondents had high postnatal mental health knowledge levels but low prenatal mental health knowledge. The least understood topic was the influence of depression or anxiety during pregnancy on child development, and only 25.4% (28/110) of participants responded with a somewhat agree or strongly agree that *“*children who have mothers with depression or anxiety during pregnancy are likely to be slower in their development than children whose mothers do not have depression or anxiety in pregnancy,*”* and >46% (51/110, 46.4%) reported that they were unsure or did not know. Findings from this study that showed that African mothers were more knowledgeable about postnatal maternal mental health than prenatal mental health are similar to both the Canadian study (in which 52% of respondents identified prenatal anxiety as “normal”) [23] and the Australian study (in which 7% of participants acknowledged the health implications of maternal anxiety and depression before childbirth) [34]. Limited knowledge of the determinants of prenatal mental health is a risk factor for poor maternal mental health after childbirth, as it contributes to the failure to recognize the role that other societal and economic factors play in maternal depression and anxiety outcomes after childbirth [20,36,37]. This failure to recognize other nonbiological factors can be a barrier to help-seeking behavior, as it implies that the prevention of maternal depression and anxiety is not possible. Strategies for improving help-seeking behavior among African mothers in Alberta should focus on improving mental health literacy, reducing stigma, and considering their desire for self-reliance.

The limited knowledge of prenatal mental health factors may also contribute to the high rates of maternal depression in African mothers in Alberta, as well as the underdetection and undertreatment of other maternal mental health problems. Improving maternal mental health knowledge before and after childbirth may have a substantial impact on the timely detection of maternal depression and anxiety and possibly on more timely help-seeking behaviors. Existing literature indicates that early and targeted interventions to improve the mental health of pregnant women have long-term benefits in reducing the risk of maternal mental health problems following childbirth and poor child development [38-40]. Although the historical focus on immigrant maternal health studies has been on diagnosing and treating depression after pregnancy, the findings from this study highlight the need for a shift toward improving African mothers’ (and immigrant mothers as a whole) understanding of mental health disorders in the prenatal period. The low level of prenatal mental health knowledge, specifically on the effects of maternal mental health outcomes on child development highlighted in this study, echoes findings from other studies indicating that knowledge of factors influencing child development is poor [41] and dismisses the importance of early life influence on long-term child health and well-being [1,23,42]. Ultimately, this study highlights that among all participants, 51.5% (53/103) knew someone who had depression or anxiety after childbirth. Of the respondents with maternal depression symptoms, >70% (18/25, 72%) knew someone who had depression or anxiety after childbirth. Personal experiences with maternal mental health play a positive role [1,23,43]. Thus, it is plausible that a consistent predictor of prenatal and postnatal knowledge is personally knowing another woman who has experienced maternal depression or anxiety.

Standardization of perinatal mental health screening (specifically postpartum depression screening) was identified as a priority in the 2017 Government of Alberta’s Valuing Mental Health Strategy [44]. Nonetheless, this is still not a required component of routine prenatal and postnatal care in Alberta. It is noteworthy that the findings of this study demonstrate the high acceptability of routine perinatal mental health screening, as 75.2% (82/109) and 82.7% (91/110) of the participants agreed that all women should be screened during the pre- and postnatal periods, respectively. This is an important finding within the framework of Alberta’s health care system; although respondents highlight the value of prenatal and postnatal mental health screening, it is currently not mandated or actively promoted within current provincial or federal perinatal policies. Findings from this study indicate that universal maternal mental health screening in the pre- and postnatal periods is acceptable among African mothers in Alberta.

In addition, perinatal mental health knowledge was a constant predictor of the acceptability of pre- and postnatal mental health screening. Respondents who were knowledgeable about the impact of maternal depression and anxiety were more likely to endorse universal screening, which highlights the dynamic interplay between perinatal mental health knowledge and perinatal mental health screening acceptability. This finding is consistent with that of known impact of health literacy on cancer screening [45,46], screening for high BMI and type 2 diabetes [47], and other forms of screening [48]. To improve the perinatal mental health knowledge of African mothers, family doctors and women’s health clinics across the province should make mental health screening in pregnancy a part of routine maternity care. This would require the prioritization of workloads, further mental health training, and a change in the attitudes and practices of health care providers. Currently, in Alberta, women’s maternity and obstetric clinics do not have resident psychiatrists or psychologists, and expectant mothers are referred elsewhere to seek services [44]. Family doctors and women’s health clinics would need support from a wider multidisciplinary team, ideally with a psychiatrist, to work together in a combined approach to develop and implement culturally appropriate guidelines to ensure that African immigrant mothers receive the best possible care in a more holistic, culturally safe, and comprehensive way.

Closely related to the low prenatal mental health knowledge among African immigrant women is the lack of targeted maternal mental health outreach. This lack of targeted mental health outreach has also been noted by South Asian and other immigrant and racialized communities in Canada [49-51]. The solution offered in these studies is to educate immigrant communities about mental illness and mental health so that the recommendation to seek timely maternal mental health care slowly emerges from the community itself. Although the participants’ knowledge of postpartum maternal mental health in this study was high, prenatal mental health education emerging directly from the African immigrant community can increase mental health literacy and mitigate the stigma surrounding seeking timely mental health care, especially before and after childbirth.

### Limitations

Similar to all cross-sectional studies, this study only provides a snapshot of the views of perinatal mental health among a self-selected sample of African mothers in Alberta at a specific time (2020). The variations in the views on perinatal mental health and acceptability of screening over time and their impact on mental health could be examined further with a longitudinal research study design. In addition, all study variables were based on self-reported data, which are subject to recall and social desirability biases. Ultimately, participants were not asked to identify their occupation, and thus we were unable to identify the views of health care professionals and if they had any impact on their responses. Furthermore, the study used nonrandom sampling (convenience sample), and as a result, the total sample size of the study might not be fully representative or generalizable to all African mothers in Alberta within 2 years after childbirth.

### Conclusions

This study highlights that the sample of African mothers in Alberta had minimal prenatal mental health knowledge and the impact of poor perinatal mental health on child development. Strengthening the African immigrant mothers’ awareness of the determinants of poor perinatal mental health knowledge and its influence on child development is crucial. Thus, future research must examine the impact of perinatal mental health literacy on screening acceptability and help-seeking patterns for maternal depression and anxiety.

The high acceptability of universal perinatal mental screening among African mothers with young infants provides a strong message to clinicians and policy makers that warrants an actionable response. The American College of Obstetricians and Gynecologists recommends routine psychosocial screening of all women seeking pregnancy evaluation or prenatal care; however, little progress has been achieved in its implementation in Canada or the United States [52]. In Canada, a new initiative, the Canadian Perinatal Mental Health Collaborative is calling for a national strategy for perinatal mental health that will provide “direction, policy, and funding for improvements to perinatal mental health care including universal screening and timely access to treatment for all individuals during preconception, pregnancy and the postpartum periods” [53]. Maternal mental health literacy programs and initiatives should capitalize on the high acceptance of universal perinatal screening among African mothers to increase prenatal mental health knowledge and screening practices.

Ultimately, the findings of this study have implications for mental health policy and delivery of mental health systems. Specifically, system-level barriers (ie, access) make it difficult to navigate the mental health system. Like other immigrant populations, African immigrants do not feel adept at navigating specialized mental health services and require targeted outreach, awareness, and educational programs to be better informed. The data showed that one of the preferences for help-seeking was to consult primary care providers for maternal mental health concerns. Thus, expanding the mental health care role of the family doctor so that they can act as the first line of defense against maternal depression and anxiety is one possible course of action for consideration. Further research is needed on how integrated care can be effectively promoted and the types of training that family doctors will require to expand their mental health care roles.

## Data Availability

The data sets generated and analyzed during this study are not publicly available because of limitations of the ethics approval application but can be made available from the corresponding author on reasonable request and with permission from the University of Lethbridge Human Participant Research Committee.
